# The London Hospital Chairman's View

**Published:** 1911-12-09

**Authors:** Sydney Holland

**Affiliations:** Chairman London Hospital. Kneesworth Hall, Royston, Herts


### THE VOLUNTARY HOSPITALS AND THE NATIONAL INSURANCE BILL.

### THE LONDON HOSPITAL CHAIRMAN'S VIEW.

SIR,—In answer to your circular-letter asking for my views on the probable effects of the National Insurance Bill, I am afraid that I differ from your opening sentence, which says that "the Insurance Bill threatens the working classes with grievous risk and much additional suffering."

I dislike very much the way in which the Bill has been introduced and rushed through Parliament, and it is obvious that even if it becomes law, which I think is doubtful, it will not long remain in its present state; there will be too strong a feeling against it.

But, with regard to its effect upon hospitals, I do not believe that the voluntary hospitals will in the end suffer financially. Every proposed reform finds its Prophets of Evil. But we all suffer much more from what does not happen than from what does. I quite think that at first employers will lessen their subscriptions or cease their subscriptions, just as all people do when taxation is increased. We have suffered from the Licensing Bill, from the Death Duties, from the Tax on Land, but the effect of these is wearing off. I remember well, when Sir W. Vernon Harcourt first introduced the Death Duties, people cried out that nobody would ever leave a penny more to charities. But they have. I have enough faith in human nature to believe that sulkiness wears off, and that people will not allow their fellow-creatures to suffer without helping them.

This answer of mine is in reply to your first two questions as to "How much the voluntary hospitals will suffer financially?" and "How much of our present accommodation may not be able to be maintained?" I will bet that not a bed is closed.

I think I ought to qualify my reply by saying that we have not in London, as you know, the same experience of large amounts being subscribed by the working classes, as provincial hospitals have. But, if it is true, and I see no reason to doubt it, that under this Bill the working-man who was insured before will be paying less for his insurance in future, and getting more for his money (though not 9d. for 4d.), then I do not see why the Bill should reduce the subscriptions of the working-men who were in, and are in, Friendly Societies. As to the subscriptions from men who have never been in Friendly Societies and now find themselves compelled to join, I doubt whether their subscriptions ever amounted to very much, because they are the less thrifty of their class. So far for in-patients.

As to out-patients, the matter is different. In theory, the only people we treat in our out-patient departments are those who, not being destitute, yet (1) cannot afford to pay a doctor, (2) those sent up for consultation opinion by a doctor, (3) those who are not satisfied with their own doctor's opinion and want a further opinion.

Under this Bill the whole of Class 1 ought to disappear, or, at any rate, all but women and children. There will be no men, other than paupers, who cannot have a medical opinion without having to pay a fee for it—and paupers ought to be referred to the Poor Law. Classes 2 and 3 will remain. I do not see what the solution of the difficulty arising from the disappearance of Class 1 will be, because a large number will be needed if we are to teach the future generations of doctors. But I prophesy that things will remain very much as they are, except that we shall have the power materially to reduce the numbers of our out-patients, which will benefit the hospitals. And I feel that in this case also we ought to be able to claim payment for those whom we treat, certainly of Classes 2 and 3, if not of Class 1.

As to your third question for my opinion, "On the eligibility or otherwise of insured persons to free hospital benefit," I cannot see that this Bill affects the question very much as regards in-patients.

The Bill provides 10s. a week for the dependents of the patient. This removes one of the greatest terrors that I meet with among hospital patients, *i.e.* that while they are ill their home is broken up, everything pawned, and the children starve.

I cannot think that a hospital can touch this money. Therefore, so far as this 10s. is concerned the eligibility of the patient is not affected. But I do feel, as the Insurance Bill provides for the payment to a doctor of a certain sum for attending the sick man, and as the patient is being attended to by the hospital, that we hospitals ought to be able to claim from the State what otherwise would have been paid to the doctor. If we succeed in getting this, the Bill will actually help hospitals.

As to your last question, "What is likely to be the consequence to the working classes of being shut out from in-patient relief at the voluntary hospitals which they at present enjoy, on the ground stated to the Hospitals Deputation by Mr. Lloyd George, *i.e.* that when they are insured they will have no right to come to the hospitals?" I do not remember Mr. Lloyd George saying that "An insured person would have no right to come to the hospital," and I feel almost sure he did not say so.*** I pointed out to him that as regards illness the medical provisions of the Bill were very good for anyone with a bad cold or an in-growing toe-nail; but that the Bill did not in any way help the working-man who was seriously ill, or required an operation to obtain what he needed, except in so far as it relieved him of his anxiety about those dependent on him. I do not remember any suggestion that an insured person should have no right to come to the hospital. It would have been absurd. There was a short discussion as to whether subscriptions could be asked for from Health Committees and Friendly Societies, where the hospitals helped members of the club, or those under the jurisdiction of the Health Committees.

The principle of obtaining payment from patients will remain unaltered by the Bill—that principle is to get all you can, and be content with nothing if you cannot get anything. In my opinion none of the working classes will be shut out from in-patient relief.—Yours faithfully,

(Sgd.) Sydney Holland,
Chairman London Hospital.

Kneesworth Hall, Royston, Herts.
December 5.

***The Chancellor of the Exchequer's Actual Words to the Deputation on July 25, 1911, were :—

"They had given power in the Bill to approved societies to contribute. The deputation would probably say that that was not sufficient; but the remedy was very largely in their own hands. Mr. Sydney Holland had said that two of the classes who used the hospital were discontented patients who were not satisfied with their doctors and the discontented doctors who sent their patients. They should say to the first class of persons, '*Are you insured?*' *If they were, then they had no right to come to the hospitals.* They should further tell the doctors that they couldn't take their burden off their shoulders.

"Mr. Holland complained that they had not legislated for hospitals. It was for the very reason that they believed in the voluntary system and wanted it to continue. If they wanted to be put upon a fund they must be put upon it under the same conditions as any other institutions, and there would have to be a certain amount of Government control over their work." *They were in a position to say to the people, "We cannot take you unless you are prepared to meet us. We are free contracting parties."*

Mr. Holland : "We cannot refuse a person who is desperately ill simply because someone declines to pay for him."

Sir William Cobbett : "Hospitals cannot refuse patients."

Mr. Lloyd George : "You cannot refuse the individual, but you can in future be in a position to insist on help from the approved societies when they get contributions from the State and enforced contributions from employers."

Mr. Lloyd George continued that "in regard to Clause 12 the suggestion had been made that in certain cases the sick pay might in one form or other be made a contribution to the hospital itself. That was worth considering, and it would undoubtedly very greatly improve the financial position of the hospital." (See The Hospital, July 29, 1911, p. 451.)

